# Supplementary material for: Global dynamics of neural mass models
Source: PLoS Comput Biol. 2023 Feb 10;19(2):e1010915. doi: 10.1371/journal.pcbi.1010915 (PMC9949652; doi:10.1371/journal.pcbi.1010915)
Supplement: S1 Text — (DOCX) [file pcbi.1010915.s001.docx]

**S1 Text. Appendix**

The complex function for the trajectory will be given by (in modulus-argument form, or equivalently in polar coordinates),

$$z_{i}(t)=R_{i}\left( t,\varepsilon,\mu\right)e^{-i(\omega_{i}t+\varphi_{i}(\left( t,\varepsilon,\mu\right)))}$$

The perturbation expansion will be given by,

$$R_{i}\left( t,\varepsilon,\mu\right)=R_{i,0,0}+\sum\varepsilon^{m}\mu^{n}R_{i,m,n}(t)$$

$$\varphi_{i}(t,\varepsilon,\mu)=\sum\varepsilon^{m}\mu^{n}\varphi_{i,m,n}(t)$$

The equation of motion will be given by,

Eq. A1

$$\left( \dot{R_{i}}\left( t,\varepsilon,\mu\right)e^{-i\left( \omega_{i}t+\varphi_{i} \right)}-iR_{i}\left( t,\varepsilon,\mu\right)\dot{\varphi_{i}}e^{-i\left( \omega_{i}t+\varphi_{i} \right)} \right)=i\varepsilon\omega_{i}\sum g_{ij}S\left( \frac{z_{j}+{z_{j}}^{*}}{2} \right)+i\mu\omega_{i}\sum g_{ij}P\left( \frac{z_{j}-{z_{j}}^{*}}{2i} \right)$$

The sigmoid function (tanh) can be expanded as,

$$S\left( \frac{z_{j}+{z_{j}}^{*}}{2} \right)= \sum_{r=1}^{\infty} A_{r}\left( \frac{z_{j}+{z_{j}}^{*}}{2} \right)^{2r-1}=\sum_{r=1}^{\infty} A_{r}\frac{\left[ R_{j}\left( t,\varepsilon,\mu\right)e^{-i\left( \omega_{j}t+\varphi_{j} \right)}+R_{j}\left( t,\varepsilon,\mu\right)e^{i\left( \omega_{j}t+\varphi_{j} \right)} \right]^{2r-1}}{2^{2r-1}}=\sum_{r=1}^{\infty} A_{r}\frac{\left( R_{j,0,0}+\sum\varepsilon^{n}\mu^{m}R_{j,n,m}(t) \right)^{2r-1}}{2^{2r-1}}e^{-i\left( 2r-1 \right)\left( \omega_{j}t+\varphi_{j} \right)}*\left[ 1+e^{i2\left( \omega_{j}t+\varphi_{j} \right)} \right]^{2r-1}=\sum_{r=1}^{\infty} A_{r}\frac{\left( R_{j,0,0}+\sum\varepsilon^{n}\mu^{m}R_{j,n,m}(t) \right)^{2r-1}}{2^{2r-1}}e^{-i\left( 2r-1 \right)\left( \omega_{j}t+\varphi_{j} \right)}*\left[ \sum_{l=0}^{2r-1} \left( \begin{matrix} 2r-1 \\ l \end{matrix} \right)e^{i2l\left( \omega_{j}t+\varphi_{j} \right)} \right]$$

$$=\sum_{r=1}^{\infty} A_{r}\frac{\left( R_{j,0,0}+\sum\varepsilon^{n}\mu^{m}R_{j,n,m}(t) \right)^{2r-1}}{2^{2r-1}}*\left[ \sum_{l=0}^{2r-1} \left( \begin{matrix} 2r-1 \\ l \end{matrix} \right)e^{-i\left( \left( 2r-1 \right)-2l \right)\left( \omega_{j}t \right)}\left[ \sum_{k=0}^{\infty} \frac{\left( -i(2r-1-2l)\varphi_{j} \right)^{k}}{k!} \right] \right]$$

The *P* function (current-to-current coupling) can be expanded as,

$$P\left( \frac{z_{j}-{z_{j}}^{*}}{2i} \right)= \sum_{r=1}^{\infty} B_{r}\left( \frac{z_{j}-{z_{j}}^{*}}{2i} \right)^{2r-1}=i\sum_{r=1}^{\infty} B_{r}\frac{\left[ R_{j}\left( t,\mu\right)e^{-i\left( \omega_{j}t+\varphi_{j} \right)}-R_{j}\left( t,\mu\right)e^{i\left( \omega_{j}t+\varphi_{j} \right)} \right]^{2r-1}}{2^{2r-1}{(-1)}^{r}}=i\sum_{r=1}^{\infty} B_{r}\frac{\left( R_{j,0}+\sum\mu^{n}R_{j,n}(t) \right)^{2r-1}}{2^{2r-1}{(-1)}^{r}}e^{-i\left( 2r-1 \right)\left( \omega_{j}t+\varphi_{j} \right)}*\left[ 1-e^{i2\left( \omega_{j}t+\varphi_{j} \right)} \right]^{2r-1}=i\sum_{r=1}^{\infty} B_{r}\frac{\left( R_{j,0}+\sum\mu^{n}R_{j,n}(t) \right)^{2r-1}}{2^{2r-1}{(-1)}^{r}}e^{-i\left( 2r-1 \right)\left( \omega_{j}t+\varphi_{j} \right)}*\left[ \sum_{l=0}^{2r-1} {(-1)}^{l}\left( \begin{matrix} 2r-1 \\ l \end{matrix} \right)e^{i2l\left( \omega_{j}t+\varphi_{j} \right)} \right]$$

$$=i\sum_{r=1}^{\infty} {(-1)}^{r}B_{r}\frac{\left( R_{j,0}+\sum\mu^{n}R_{j,n}(t) \right)^{2r-1}}{2^{2r-1}}*\left[ \sum_{l=0}^{2r-1} {(-1)}^{l}\left( \begin{matrix} 2r-1 \\ l \end{matrix} \right)e^{-i\left( \left( 2r-1 \right)-2l \right)\left( \omega_{j}t \right)}\left[ \sum_{k=0}^{\infty} \frac{\left( -i(2r-1-2l)\varphi_{j} \right)^{k}}{k!} \right] \right]$$

Expanding the LHS of Eq A1.

Eq. A2

$$\left( \dot{R_{i}}\left( t,\varepsilon,\mu\right)e^{-i\left( \omega_{i}t+\varphi_{i} \right)}-iR_{i}\left( t,\varepsilon,\mu\right)\dot{\varphi_{i}}e^{-i\left( \omega_{i}t+\varphi_{i} \right)} \right)=\left[ \sum_{k=0}^{\infty} \frac{\left( -i\varphi_{i} \right)^{k}}{k!} \right]\left( \sum\varepsilon^{n}\mu^{m}\dot{R}_{i,n,m}\left( t \right) \right)e^{-i\omega_{i}t}-i\left( R_{i,0,0}+\sum\varepsilon^{n}\mu^{m}R_{i,n,m}\left( t \right) \right)*\left( \sum\varepsilon^{n}\mu^{m}\dot{\varphi}_{i,n,m}(t) \right)\left[ \sum_{k=0}^{\infty} \frac{\left( -i\varphi_{i} \right)^{k}}{k!} \right]e^{-i\omega_{i}t}$$

The RHS of Eq. A1, after multiplying with $e^{i\omega_{i}t}$, will be given by,

Eq. A3

$$i\omega_{i}\varepsilon e^{i\omega_{i}t}\sum g_{ij}\sum_{r=1}^{\infty} A_{r}\frac{\left( R_{j,0,0}+\sum\varepsilon^{n}\mu^{m}R_{j,n,m}(t) \right)^{2r-1}}{2^{2r-1}}*\left[ \sum_{l=0}^{2r-1} \left( \begin{matrix} 2r-1 \\ l \end{matrix} \right)e^{-i\left( \left( 2r-1 \right)-2l \right)\left( \omega_{j}t \right)}\left[ \sum_{k=0}^{\infty} \frac{\left( -i(2r-1-2l)\varphi_{j} \right)^{k}}{k!} \right] \right]+i\omega_{i}\mu e^{i\omega_{i}t}\sum g_{ij}\sum_{r=1}^{\infty} {(-1)}^{r}B_{r}\frac{\left( R_{j,0,0}+\sum\mu^{n}R_{j,0,n}(t) \right)^{2r-1}}{2^{2r-1}}*\left[ \sum_{l=0}^{2r-1} {(-1)}^{l}\left( \begin{matrix} 2r-1 \\ l \end{matrix} \right)e^{-i\left( \left( 2r-1 \right)-2l \right)\left( \omega_{j}t \right)}\left[ \sum_{k=0}^{\infty} \frac{\left( -i(2r-1-2l)\varphi_{j} \right)^{k}}{k!} \right] \right]$$

**S1.1. Potential-to-current coupling**

We will investigate the effect of potential-to-current coupling by keeping $\mu=0$, keeping only the *S*-function.

*S1.1.1 Expanding the* $\varepsilon^{1}$ *term*

Keeping only $\varepsilon^{1}$terms for the LHS (Eq. A2) will give,

$$\varepsilon^{1}\dot{R}_{i,1,0}\left( t \right)-iR_{i,0,0}\varepsilon^{1}\dot{\varphi}_{i,1,0}(t)$$

Keeping only $\varepsilon^{1}$terms for the RHS (Eq. A3) will give,

$$i\varepsilon\omega_{i}\sum g_{ij}\sum_{r=1}^{\infty} A_{r}\frac{{R_{j,0,0}}^{2r-1}}{2^{2r-1}}\left[ \sum_{l=0}^{2r-1} \left( \begin{matrix} 2r-1 \\ l \end{matrix} \right)e^{-i\left( \left( 2r-1 \right)-2l \right)\left( \omega_{j}t \right)+i\omega_{i}t} \right]$$

Equating these terms will give,

$$\dot{R}_{i,1,0}\left( t \right)-iR_{i,0,0}\dot{\varphi}_{i,1,0}\left( t \right)=i\omega_{i}\sum g_{ij}\sum_{r=1}^{\infty} A_{r}\frac{{R_{j,0,0}}^{2r-1}}{2^{2r-1}}e^{-i\left( 2r-1 \right)\left( \omega_{j}t \right)+i\omega_{i}t}\left[ \sum_{l=0}^{2r-1} \left( \begin{matrix} 2r-1 \\ l \end{matrix} \right)e^{i2l\left( \omega_{j}t \right)} \right]\equiv W_{i,1,0}$$

We can then calculate the following,

$$\dot{R}_{i,1,0}\left( t \right)=\frac{W_{i,1,0}+{W_{i,1,0}}^{*}}{2}$$

$$\dot{\varphi}_{i,1,0}\left( t \right)=i\frac{W_{i,1,0}-{W_{i,1,0}}^{*}}{2R_{i,0,0}}$$

Integrating the terms will give,

Eq. A4

$$R_{i,1,0}\left( t \right)=\int_{0}^{t} \dot{R}_{i,1,0}\left( t' \right)=\int_{0}^{t} \frac{W_{i,1,0}+{W_{i,1,0}}^{*}}{2}$$

Eq. A5

$$\varphi_{i,1,0}\left( t \right)=\int_{0}^{t} \dot{\varphi}_{i,1,0}\left( t' \right)=i\int_{0}^{t} \frac{W_{i,1,0}-{W_{i,1,0}}^{*}}{2R_{i,0,0}}$$

The integral on *W* will be given by,

$$\int_{0}^{t} W_{i,1,0}=i\omega_{i}\sum g_{ij}\sum_{r=1}^{\infty} A_{r}\frac{{R_{j,0,0}}^{2r-1}}{2^{2r-1}}\left[ \sum_{l=0}^{2r-1} \left( \begin{matrix} 2r-1 \\ l \end{matrix} \right)\int_{0}^{t} e^{i(\omega_{i}t-\left( 2r-1-2l \right)\left( \omega_{j}t \right))} \right]$$

If $i\neq j$, we will get the following,

$$\int_{0}^{t} W_{i,1,0}=\omega_{i}\sum g_{ij}\sum_{r=1}^{\infty} A_{r}\frac{{R_{j,0,0}}^{2r-1}}{2^{2r-1}}\left[ \sum_{l=0}^{2r-1} \left( \begin{matrix} 2r-1 \\ l \end{matrix} \right)\frac{(e^{i\left( \omega_{i}-\left( 2r-1-2l \right)\omega_{j} \right)t}-1)}{(\omega_{i}-\left( 2r-1-2l \right)\omega_{j})} \right]$$

Eq. 4 and 5 will then give the following,

$$R_{i,1,0}\left( t \right)=\int_{0}^{t} \frac{W_{i,1,0}+{W_{i,1,0}}^{*}}{2}=\frac{\omega_{i}}{2}\sum g_{ij}\sum_{r=1}^{\infty} A_{r}\frac{{R_{j,0,0}}^{2r-1}}{2^{2r-1}}*\left[ \sum_{l=0}^{2r-1} \left( \begin{matrix} 2r-1 \\ l \end{matrix} \right)\frac{\left( e^{i\left( \omega_{i}-\left( 2r-1-2l \right)\omega_{j} \right)t}+e^{-i\left( \omega_{i}-\left( 2r-1-2l \right)\omega_{j} \right)t} \right)-2}{(\omega_{i}-\left( 2r-1-2l \right)\omega_{j})} \right]$$

$$\varphi_{i,1,0}\left( t \right)=i\int_{0}^{t} \frac{W_{i,1,0}-{W_{i,1,0}}^{*}}{2R_{i,0,0}}=\frac{i\omega_{i}}{2R_{i,0,0}}\sum g_{ij}\sum_{r=1}^{\infty} A_{r}\frac{{R_{j,0,0}}^{2r-1}}{2^{2r-1}}**\left[ \sum_{l=0}^{2r-1} \left( \begin{matrix} 2r-1 \\ l \end{matrix} \right)\frac{(e^{i\left( \omega_{i}-\left( 2r-1-2l \right)\omega_{j} \right)t}-e^{-i\left( \omega_{i}-\left( 2r-1-2l \right)\omega_{j} \right)t})}{(\omega_{i}-\left( 2r-1-2l \right)\omega_{j})} \right]$$

We can them see that the following holds,

$$R_{i,1,0}\left( T \right)=\varphi_{i,1,0}\left( T \right)=0$$

If i=j, we will get the following,

$$\int_{0}^{t} W_{i,1,0}=-\omega_{i}g_{ii}\sum_{r=1}^{\infty} A_{r}\frac{{R_{i,0,0}}^{2r-1}}{2^{2r-1}}\sum_{l=0, l\neq r-1}^{2r-1} \left( \begin{matrix} 2r-1 \\ l \end{matrix} \right)\frac{e^{-i\left( \left( 2r-2 \right)-2l \right)\omega_{i}t}-1}{\left( \left( 2r-2 \right)-2l \right)\omega_{i}t}+i\omega_{i}g_{ii}\sum_{r=1}^{\infty} A_{r}\frac{{R_{i,0,0}}^{2r-1}}{2^{2r-1}}\left( \begin{matrix} 2r-1 \\ r-1 \end{matrix} \right)t$$

We thus have, where 1^st^ and 2^nd^ indicate the terms on the RHS in the preceding eq.,

$$2R_{i,1,0}=1st+ conjugate$$

$$-2iR_{i,0,0}\varphi_{i,1,0}=\left( 1st+2nd \right)-conjugate$$

We can now see that the following holds,

$$\frac{\varphi_{i,1,0}\left( T \right)}{T}=-\frac{\omega_{i}}{2R_{i,0,0}}g_{ii}\sum_{r=1}^{\infty} A_{r}\frac{{R_{i,0,0}}^{2r-1}}{2^{2r-1}}\left( \begin{matrix} 2r-1 \\ r-1 \end{matrix} \right)$$

$$R_{i,1,0}\left( T \right)=0$$

In summary the topology of the phase space dynamics is stable for perturbations of first order in $\varepsilon$. The effect of the perturbation will be a change in the speed of rotation $\omega_{i}$.

$$\bar{\omega_{i}}=\omega_{i}\left( 1-\frac{1}{2R_{i,0,0}}g_{ii}\sum_{r=1}^{\infty} A_{r}\frac{{R_{j,0,0}}^{2r-1}}{2^{2r-1}}\left( \begin{matrix} 2r-1 \\ r-1 \end{matrix} \right) \right)$$

*S1.1.2 Expanding the* $\varepsilon^{2}$ *term*

Keeping only $\varepsilon^{2}$ terms for the LHS of Eq. A1 will give,

$$LHS=\left[ \left( \dot{R}_{i,2,0}-iR_{i,0,0}\dot{\varphi}_{i,2,0} \right)+\frac{d}{dt}\left[ -iR_{i,1,0}\varphi_{i,1,0}-\frac{R_{i,0,0}{\varphi_{i,1,0}}^{2}}{2} \right] \right]e^{-i\omega_{i}t}$$

Integrating this term after multiplying with $e^{i\omega_{i}t}$ will give,

$$\left( R_{i,2,0}(t)-iR_{i,0,0}\varphi_{i,2,0}(t) \right)-iR_{i,1,0}\varphi_{i,1,0}-\frac{R_{i,0,0}{\varphi_{i,1,0}}^{2}}{2}$$

The RHS of Eq. A1 (Eq A3) after multiplying with $e^{i\omega_{i}t}$ will give,

$$=i\varepsilon^{1}\omega_{i}\sum g_{ij}\sum_{r=1}^{\infty} A_{r}\frac{\left( R_{j,0,0}+\sum\varepsilon^{n}\mu^{m}R_{j,n,m}(t) \right)^{2r-1}}{2^{2r-1}}*\left[ \sum_{l=0}^{2r-1} \left( \begin{matrix} 2r-1 \\ l \end{matrix} \right)e^{-i\left( \left( 2r-1 \right)-2l \right)\left( \omega_{j}t \right)+i\omega_{i}t}\left[ \sum_{k=0}^{\infty} \frac{\left( -i(2r-1-2l)\varphi_{j} \right)^{k}}{k!} \right] \right]$$

Keeping only $\varepsilon^{2}$ terms will give,

Eq. A6

$$=i\omega_{i}\sum g_{ij}\sum_{r=1}^{\infty} A_{r}\frac{{R_{j,0,0}}^{2r-1}}{2^{2r-1}}*\left[ \sum_{l=0}^{2r-1} \left( \begin{matrix} 2r-1 \\ l \end{matrix} \right)e^{-i\left( \left( 2r-1 \right)-2l \right)\left( \omega_{j}t \right)+i\omega_{i}t}\left( -i(2r-1-2l)\varphi_{j,1,0} \right) \right]+i\omega_{i}\sum g_{ij}\sum_{r=1}^{\infty} A_{r}\frac{(2r-1){R_{j,0,0}}^{2r-2}R_{j,1,0}}{2^{2r-1}}*\left[ \sum_{l=0}^{2r-1} \left( \begin{matrix} 2r-1 \\ l \end{matrix} \right)e^{-i\left( \left( 2r-1 \right)-2l \right)\left( \omega_{j}t \right)+i\omega_{i}t} \right]$$

Note that,

$$\varphi_{j,1,0}=\frac{i\omega_{j}}{2R_{i,0,0}}\sum_{j\neq k} g_{jk}\ldots+g_{jj}t\ldots$$

$$R_{j,1,0}=\frac{\omega_{j}}{2}\sum g_{jk}\ldots$$

Both functions will have a sum of products of exponentials. If none of the indices; i, j or k are equal, integrating this over a period T will not result in a net change. I.e. the cross terms will not contribute to a change in location of the trajectories over one full period T. The other four possible situations will be,

$$i=j\neq k$$

$$i=k\neq j$$

$$j=k\neq i$$

$$i=j=k$$

*If* $i=j\neq k$

The first term in Eq. A6 will contain the following term,

$$e^{\pm i\left( \omega_{i}-\left( 2r-1-2l \right)\omega_{k} \right)t}$$

This term will not contribute to a change when integrated over a full cycle (t=T). The second term will contain the following terms,

$$e^{\pm i\left( \omega_{i}-\left( 2r-1-2l \right)\omega_{k} \right)t} and 1$$

The constant term will contribute to a change in the trajectory when integrated over a full cycle but will not change *R* but only $\varphi$ as the change will be imaginary. A similar situation will apply when $i=k\neq j$*.*

*If* $j=k\neq i$

The first term in Eq A6 will contain the following term,

$$\ldots e^{\pm i\left( \omega_{i}-\left( 2r-1-2l \right)\omega_{j} \right)t}g_{jj}t\ldots$$

When this term is integrated using partial integration over the full cycle there will be an imaginary component at T, which will not contribute to a change in *R*. The terms from the second part will all have the following term,

$$e^{-i\omega_{i}t}$$

And will hence not have a non-zero contribution over a full cycle (t=T).

*If* $j=k=i$

The 2^nd^ term in Eq. A6 will have an imaginary contribution and hence not change *R*. The 1^st^ term will be given by,

$$=i\omega_{i}g_{ii}\sum_{r=1}^{\infty} A_{r}\frac{{R_{i,0,0}}^{2r-1}}{2^{2r-1}}*\left[ \sum_{l=0}^{2r-1} \left( \begin{matrix} 2r-1 \\ l \end{matrix} \right)e^{-i\left( \left( 2r-2 \right)-2l \right)\left( \omega_{i}t \right)}\left( -i(2r-1-2l)\varphi_{i,1,0} \right) \right]$$

Where,

Eq. A7

$$\varphi_{i,1,0}\left( t \right)=-i\frac{\omega_{i}g_{ii}}{2R_{i,0,0}}\sum_{s=1}^{\infty} A_{s}\frac{{R_{i,0,0}}^{2s-1}}{2*2^{2s-1}}\sum_{m=0, m\neq s-1}^{2s-1} \left( \begin{matrix} 2s-1 \\ m \end{matrix} \right)\frac{e^{-i\left( \left( 2s-2 \right)-2m \right)\omega_{i}t}-e^{i\left( \left( 2s-2 \right)-2m \right)\omega_{i}t}}{\left( \left( 2s-2 \right)-2m \right)\omega_{i}t}-\frac{\omega_{i}g_{ii}}{R_{i,0,0}}\sum_{s=1}^{\infty} A_{s}\frac{{R_{i,0,0}}^{2s-1}}{2^{2s-1}}\left( \begin{matrix} 2s-1 \\ s-1 \end{matrix} \right)t$$

The last term in Eq. 7 will give a real contribution at T if,

$$\left( 2r-2 \right)-2l=0$$

We then have for the 1st term in Eq. 6,

$$\omega_{i}g_{ii}\sum_{r=1}^{\infty} A_{r}\frac{{R_{i,0,0}}^{2r-1}}{2^{2r-1}}\left( \begin{matrix} 2r-1 \\ r-1 \end{matrix} \right)\varphi_{i,1,0}$$

Integrating this term will give,

$$-\omega_{i}g_{ii}\sum_{r=1}^{\infty} A_{r}\frac{{R_{i,0,0}}^{2r-1}}{2^{2r-1}}\left( \begin{matrix} 2r-1 \\ r-1 \end{matrix} \right)\frac{\omega_{i}g_{ii}}{R_{i,0,0}}\sum_{s=1}^{\infty} A_{s}\frac{{R_{i,0,0}}^{2s-1}}{2*2^{2s-1}}\left( \begin{matrix} 2s-1 \\ s-1 \end{matrix} \right)T^{2}=-\frac{R_{i,0,0}{\varphi_{i,1,0}(T)}^{2}}{2}$$

This term will cancel the non-zero term on the LHS of Eq. A2.

$$-\frac{R_{i,0,0}{\varphi_{i,1,0}}^{2}}{2}$$

The 1st term in Eq. 7 will have an imaginary contribution and hence not change R. We have thus shown that the topology of the phase-space trajectories are stable for perturbations upto 2^nd^ order.

**S1.2. Spike rate coupling**

We will now investigate the effect of the spike rate coupling, i.e we will keep $\varepsilon=0$ and analyse the effect of $\mu$ on the dynamics.

*S1.2.1 Expanding* $\mu^{1}$*term,*

Keeping only $\mu^{1}$terms for the LHS of Eq. A1 (Eq. A2) will give,

$$\mu^{1}\dot{R}_{i,0,1}\left( t \right)e^{-i\omega_{i}t}-iR_{i,0,0}\mu^{1}\dot{\varphi}_{i,0,1}(t)e^{-i\omega_{i}t}$$

Keeping only $\mu^{1}$terms for the RHS of Eq. A1 (Eq. A3) will give,

$$-\mu{\omega_{i}}^{2}\sum g_{ij}\sum_{r=1}^{\infty} {(-1)}^{r}B_{r}\frac{{R_{j,0,0}}^{2r-1}}{2^{2r-1}}*\left[ \sum_{l=0}^{2r-1} {(-1)}^{l}\left( \begin{matrix} 2r-1 \\ l \end{matrix} \right)e^{-i\left( \left( 2r-1 \right)-2l \right)\left( \omega_{j}t \right)} \right]$$

Equating these terms will give,

$$\dot{R}_{i,0,1}\left( t \right)e^{-i\omega_{i}t}-iR_{i,0,0}\dot{\varphi}_{i,0,1}\left( t \right)e^{-i\omega_{i}t}=-\omega_{i}\sum g_{ij}\sum_{r=1}^{\infty} {(-1)}^{r}B_{r}\frac{{R_{j,0,0}}^{2r-1}}{2^{2r-1}}*\left[ \sum_{l=0}^{2r-1} {(-1)}^{l}\left( \begin{matrix} 2r-1 \\ l \end{matrix} \right)e^{-i\left( \left( 2r-1 \right)-2l \right)\left( \omega_{j}t \right)} \right]$$

$$\dot{R}_{i,0,1}\left( t \right)-iR_{i,0,0}\dot{\varphi}_{i,0,1}\left( t \right)=-\omega_{i}\sum g_{ij}\sum_{r=1}^{\infty} {(-1)}^{r}B_{r}\frac{{R_{j,0,0}}^{2r-1}}{2^{2r-1}}*\left[ \sum_{l=0}^{2r-1} {(-1)}^{l}\left( \begin{matrix} 2r-1 \\ l \end{matrix} \right)e^{-i\left( \left( 2r-1 \right)-2l \right)\left( \omega_{j}t \right)+i\omega_{i}t} \right]=W_{i,0,1}$$

$$\dot{R}_{i,0,1}\left( t \right)=\frac{W_{i,0,1}+{W_{i,0,1}}^{*}}{2}$$

$$\dot{\varphi}_{i,0,1}\left( t \right)=i\frac{W_{i,0,1}-{W_{i,0,1}}^{*}}{2R_{i,0,0}}$$

Integrating the terms will give,

$$R_{i,0,1}\left( t \right)=\int_{0}^{t} \dot{R}_{i,0,1}\left( t' \right)=\int_{0}^{t} \frac{W_{i,0,1}+{W_{i,0,1}}^{*}}{2}$$

$$\varphi_{i,0,1}\left( t \right)=\int_{0}^{t} \dot{\varphi}_{i,0,1}\left( t' \right)=i\int_{0}^{t} \frac{W_{i,0,1}-{W_{i,0,1}}^{*}}{2R_{i,0,0}}$$

The integral on *W* will be given by,

$$\int_{0}^{t} W_{i,1,0}=-\omega_{i}\sum g_{ij}\sum_{r=1}^{\infty} {(-1)}^{r}B_{r}\frac{{R_{j,0,0}}^{2r-1}}{2^{2r-1}}*\left[ \sum_{l=0}^{2r-1} {(-1)}^{l}\left( \begin{matrix} 2r-1 \\ l \end{matrix} \right)\int e^{-i\left( \left( 2r-1 \right)-2l \right)\left( \omega_{j}t \right)+i\omega_{i}t} \right]$$

If $i\neq j$, we will get the following,

$$\int_{0}^{t} W_{i,0,1}=i\omega_{i}\sum g_{ij}\sum_{r=1}^{\infty} {(-1)}^{r}B_{r}\frac{{R_{j,0,0}}^{2r-1}}{2^{2r-1}}\left[ \sum_{l=0}^{2r-1} {(-1)}^{l}\left( \begin{matrix} 2r-1 \\ l \end{matrix} \right)\frac{(e^{i\left( \omega_{i}-\left( 2r-1-2l \right)\omega_{j} \right)t}-1)}{(\omega_{i}-\left( 2r-1-2l \right)\omega_{j})} \right]$$

$$R_{i,0,1}\left( t \right)=\int_{0}^{t} \frac{W_{i,0,1}+{W_{i,0,1}}^{*}}{2}=i\omega_{i}\sum g_{ij}\sum_{r=1}^{\infty} {(-1)}^{r}B_{r}\frac{{R_{j,0,0}}^{2r-1}}{2^{2r}}\left[ \sum_{l=0}^{2r-1} {(-1)}^{l}\left( \begin{matrix} 2r-1 \\ l \end{matrix} \right)\frac{(e^{i\left( \omega_{i}-\left( 2r-1-2l \right)\omega_{j} \right)t}-e^{-i\left( \omega_{i}-\left( 2r-1-2l \right)\omega_{j} \right)t})}{(\omega_{i}-\left( 2r-1-2l \right)\omega_{j})} \right]$$

$$\varphi_{i,0,1}\left( t \right)=i\int_{0}^{t} \frac{W_{i,0,1}-{W_{i,0,1}}^{*}}{2R_{i,0,0}}=i\omega_{i}\sum g_{ij}\sum_{r=1}^{\infty} {(-1)}^{r}B_{r}\frac{{R_{j,0,0}}^{2r-1}}{2^{2r}R_{i,0,0}}\left[ \sum_{l=0}^{2r-1} {(-1)}^{l}\left( \begin{matrix} 2r-1 \\ l \end{matrix} \right)\frac{(ie^{i\left( \omega_{i}-\left( 2r-1-2l \right)\omega_{j} \right)t}+ie^{-i\left( \omega_{i}-\left( 2r-1-2l \right)\omega_{j} \right)t}-2i)}{(\omega_{i}-\left( 2r-1-2l \right)\omega_{j})} \right]$$

We have shown that the off diagonal terms ($i\neq j$) do not change the topology of the phase space structure upto first order, i.e.,

$$R_{i,0,1}\left( T \right)=\varphi_{i,0,1}\left( T \right)=0$$

If i=j we will have,

$$\int_{0}^{t} W_{i,0,1}=-\omega_{i}g_{ii}\sum_{r=1}^{\infty} {(-1)}^{r}B_{r}\frac{{R_{i,0,0}}^{2r-1}}{2^{2r-1}}*\left[ \sum_{l=0}^{2r-1} {(-1)}^{l}\left( \begin{matrix} 2r-1 \\ l \end{matrix} \right)\int e^{-i\left( \left( 2r-2 \right)-2l \right)\left( \omega_{i}t \right)} \right]$$

$$=-t\omega_{i}g_{ii}\sum_{r=1}^{\infty} \left( -1 \right)^{r}\left( -1 \right)^{r-1}B_{r}\frac{{R_{i,0,0}}^{2r-1}}{2^{2r-1}}\left( \begin{matrix} 2r-1 \\ r-1 \end{matrix} \right)-ig_{ii}\sum_{r=1}^{\infty} {(-1)}^{r}B_{r}\frac{{R_{i,0,0}}^{2r-1}}{2^{2r-1}}*\left[ \sum_{l\neq r-1,l=0}^{2r-1} \left( -1 \right)^{l}\left( \begin{matrix} 2r-1 \\ l \end{matrix} \right)\frac{e^{-i\left( \left( 2r-2 \right)-2l \right)\left( \omega_{i}t \right)}-1}{\left( \left( 2r-2 \right)-2l \right)} \right]$$

$$R_{i,1}\left( T \right)=\mu T\omega_{i}g_{ii}\sum_{r=1}^{\infty} B_{r}\frac{{R_{i,0,0}}^{2r-1}}{2^{2r-1}}\left( \begin{matrix} 2r-1 \\ r-1 \end{matrix} \right)$$

Hence, self-interaction terms create changes in the R coordinate as the flow completes full cycles. The rate of change in *R* can be estimated as,

$$\frac{dR_{i}}{dt}=\frac{\delta R_{i}}{T}=\frac{\mu R_{i,0,1}\left( T \right)}{T}=\mu\omega_{i}g_{ii}\sum_{r=1}^{\infty} B_{r}\frac{{R_{i,0,0}}^{2r-1}}{2^{2r-1}}\left( \begin{matrix} 2r-1 \\ r-1 \end{matrix} \right)$$

The zeroes of the above function will correspond to stationary points or limits cycles. E.g.

$$-\mu\omega_{i}g_{ii}\left( 4R_{i,0,0}-5{R_{i,0,0}}^{3}+{R_{i,0,0}}^{5} \right)$$

The above equation will have stationary points or limit cycles at 0,1 and 2. The corresponding value of $B_{r}$ will be given by,

$$B_{r}=\{8,-\frac{40}{3},\frac{16}{5}\}$$

N-stable limit cycles and a stationary point at the origin could be given using the following equation,

$$-\mu\omega_{i}g_{ii}R_{i,0,0}{{(R}_{i,0,0}}^{2}-{a_{1}}^{2})\ldots({R_{i,0,0}}^{2}-{a_{2N}}^{2})$$

$$=-\mu\omega_{i}g_{ii}\left[ {(-1)}^{2N}R_{i,0,0}\prod_{i=1}^{2N} {a_{i}}^{2}+{(-1)}^{2N-1}{R_{i,0,0}}^{3}\sum_{j=1}^{2N} \frac{1}{{a_{j}}^{2}}\prod_{i=1}^{2N} {a_{i}}^{2}+\ldots+\left( -1 \right)^{2N-n}{R_{i,0,0}}^{1}{R_{i,0,0}}^{2n}\sum_{1\leq j_{1}<\ldots<a_{j_{n}}\leq2N} \frac{1}{{a_{j_{1}}}^{2}\ldots{a_{j_{n}}}^{2}}\prod_{i=1}^{2N} {a_{i}}^{2}+\ldots+{R_{i,0,0}}^{4N+1} \right]$$

The corresponding values of $B_{r}$will be given by,

$$B_{n+1}=\frac{\left( -1 \right)^{2N-n}2^{2n+1}{R_{i,0}}^{2n+1}}{\left( \begin{matrix} 2n+1 \\ n \end{matrix} \right)}\prod_{i=1}^{2N} {a_{i}}^{2}\sum_{1\leq j_{1}<\ldots<a_{j_{n}}\leq2N}^{P(2N,n)} \frac{1}{{a_{j_{1}}}^{2}\ldots{a_{j_{n}}}^{2}}$$

We have used $P(2N,n)$ to indicate that the sum is taken over all combinations of n index points from a set of 2N index points. The dynamics of *R* can be written as a potential flow.

$$\frac{dR_{i}}{dt}=-\frac{dU}{dR_{i}}$$

$$U=-\mu\omega_{i}g_{ii}\sum_{r=1}^{\infty} B_{r}\frac{{R_{i,0,0}}^{2r}}{r2^{2r}}\left( \begin{matrix} 2r-1 \\ r-1 \end{matrix} \right)$$

*S1.2.2 Expanding the* $\mu^{2}$ *term*

Keeping only $\mu^{2}$ terms gives for the LHS of Eq. A1 (Eq. A2),

$$LHS=\left[ \left( \dot{R}_{i,0,2}-iR_{i,0,0}\dot{\varphi}_{i,0,2} \right)+\frac{d}{dt}\left[ -iR_{i,0,1}\varphi_{i,0,1}-\frac{R_{i,0,0}{\varphi_{i,0,1}}^{2}}{2} \right] \right]e^{-i\omega_{i}t}$$

Integrating this term after multiplying with $e^{i\omega_{i}t}$ will give,

$$\left. \left[ \left( R_{i,2}(t)-iR_{i,0,0}\varphi_{i,0,2}(t) \right)-iR_{i,0,1}\varphi_{i,0,1}-\frac{R_{i,0,0}{\varphi_{i,0,1}}^{2}}{2} \right] \right|_{0}^{t}$$

The RHS of Eq A3 will give after multiplying with $e^{i\omega_{i}t}$,

$$=-\mu^{1}\omega_{i}\sum g_{ij}\sum_{r=1}^{\infty} {(-1)}^{r}B_{r}\frac{\left( R_{j,0,0}+\sum\mu^{n}R_{j,0,n}(t) \right)^{2r-1}}{2^{2r-1}}*\left[ \sum_{l=0}^{2r-1} {(-1)}^{l}\left( \begin{matrix} 2r-1 \\ l \end{matrix} \right)e^{-i\left( \left( 2r-1 \right)-2l \right)\left( \omega_{j}t \right)+i\omega_{i}t}\left[ \sum_{k=0}^{\infty} \frac{\left( -i(2r-1-2l)\varphi_{j} \right)^{k}}{k!} \right] \right]$$

Keeping only $\mu^{2}$ terms will give,

Eq. A8

$$=-\omega_{i}\sum g_{ij}\sum_{r=1}^{\infty} {(-1)}^{r}B_{r}\frac{{R_{j,0,0}}^{2r-1}}{2^{2r-1}}*\left[ \sum_{l=0}^{2r-1} {(-1)}^{l}\left( \begin{matrix} 2r-1 \\ l \end{matrix} \right)e^{-i\left( \left( 2r-1 \right)-2l \right)\left( \omega_{j}t \right)+i\omega_{i}t}*\left( -i(2r-1-2l)\varphi_{j,0,1} \right) \right]-\omega_{i}\sum g_{ij}\sum_{r=1}^{\infty} {(-1)}^{r}B_{r}\frac{(2r-1){R_{j,0,0}}^{2r-2}R_{j,0,1}}{2^{2r-1}}*\left[ \sum_{l=0}^{2r-1} {(-1)}^{l}\left( \begin{matrix} 2r-1 \\ l \end{matrix} \right)e^{-i\left( \left( 2r-1 \right)-2l \right)\left( \omega_{j}t \right)+i\omega_{i}t} \right]$$

The first order components are given by (if $i\neq j$),

$$R_{i,0,1}\left( t \right)=i\omega_{i}\sum g_{ij}\sum_{r=1}^{\infty} {(-1)}^{r}B_{r}\frac{{R_{j,0,0}}^{2r-1}}{2^{2r}}\left[ \sum_{l=0}^{2r-1} {(-1)}^{l}\left( \begin{matrix} 2r-1 \\ l \end{matrix} \right)\frac{(e^{i\left( \omega_{i}-\left( 2r-1-2l \right)\omega_{j} \right)t}-e^{-i\left( \omega_{i}-\left( 2r-1-2l \right)\omega_{j} \right)t})}{(\omega_{i}-\left( 2r-1-2l \right)\omega_{j})} \right]$$

$$\varphi_{i,0,1}\left( t \right)=\omega_{i}\sum g_{ij}\sum_{r=1}^{\infty} {(-1)}^{r}B_{r}\frac{{R_{j,0,0}}^{2r-1}}{2^{2r}R_{i,0,0}}\left[ \sum_{l=0}^{2r-1} {(-1)}^{l}\left( \begin{matrix} 2r-1 \\ l \end{matrix} \right)\frac{(e^{i\left( \omega_{i}-\left( 2r-1-2l \right)\omega_{j} \right)t}+e^{-i\left( \omega_{i}-\left( 2r-1-2l \right)\omega_{j} \right)t}-2)}{(\omega_{i}-\left( 2r-1-2l \right)\omega_{j})} \right]$$

The diagonal component (if $i=j$ will be given by,

$$R_{i,0,1}\left( t \right)=-t\omega_{i}g_{ii}\sum_{r=1}^{\infty} \left( -1 \right)^{r}\left( -1 \right)^{r-1}B_{r}\frac{{R_{i,0,0}}^{2r-1}}{2^{2r-1}}\left( \begin{matrix} 2r-1 \\ r-1 \end{matrix} \right)-ig_{ii}\sum_{r=1}^{\infty} {(-1)}^{r}B_{r}\frac{{R_{i,0,0}}^{2r-1}}{2^{2r-1}}*\left[ \sum_{l\neq r-1,l=0}^{2r-1} \left( -1 \right)^{l}\left( \begin{matrix} 2r-1 \\ l \end{matrix} \right)\frac{e^{-i\left( \left( 2r-2 \right)-2l \right)\left( \omega_{i}t \right)}-e^{i\left( \left( 2r-2 \right)-2l \right)\left( \omega_{i}t \right)}}{2\left( \left( 2r-2 \right)-2l \right)} \right]$$

$$\varphi_{i,0,1}\left( t \right)=g_{ii}\sum_{r=1}^{\infty} {(-1)}^{r}B_{r}\frac{{R_{i,0,0}}^{2r-2}}{2^{2r-1}}*\left[ \sum_{l\neq r-1,l=0}^{2r-1} \left( -1 \right)^{l}\left( \begin{matrix} 2r-1 \\ l \end{matrix} \right)\frac{e^{-i\left( \left( 2r-2 \right)-2l \right)\left( \omega_{i}t \right)}+e^{i\left( \left( 2r-2 \right)-2l \right)\left( \omega_{i}t \right)}-2}{2\left( \left( 2r-2 \right)-2l \right)} \right]$$

The following conditions will need to be used to expand the equations for the 2^nd^ order contribution to $R$ and $\varphi$ (the indices will run over 3 subpopulations of neurons, i, j and k):

1. $i=j=k$

We will then have the following for the RHS,

Eq. A9

$$=-\omega_{i}g_{ii}\sum_{r=1}^{\infty} {(-1)}^{r}B_{r}\frac{{R_{i,0,0}}^{2r-1}}{2^{2r-1}}*\left[ \sum_{l=0}^{2r-1} {(-1)}^{l}\left( \begin{matrix} 2r-1 \\ l \end{matrix} \right)e^{-i\left( \left( 2r-2 \right)-2l \right)\left( \omega_{i}t \right)}*\left( -i(2r-1-2l)\varphi_{i,0,1} \right) \right]-\omega_{i}g_{ii}\sum_{r=1}^{\infty} {(-1)}^{r}B_{r}\frac{(2r-1){R_{i,0,0}}^{2r-2}R_{i,0,1}}{2^{2r-1}}*\left[ \sum_{l=0}^{2r-1} {(-1)}^{l}\left( \begin{matrix} 2r-1 \\ l \end{matrix} \right)e^{-i\left( \left( 2r-2 \right)-2l \right)\left( \omega_{i}t \right)} \right]$$

The first term in Eq. A9 will give the following,

$$-\omega_{i}g_{ii}\sum_{r=1}^{\infty} {(-1)}^{r}B_{r}\frac{{R_{i,0,0}}^{2r-1}}{2^{2r-1}}*\left[ \sum_{l=0}^{2r-1} {(-1)}^{l}\left( \begin{matrix} 2r-1 \\ l \end{matrix} \right)e^{-i\left( \left( 2r-2 \right)-2l \right)\left( \omega_{i}t \right)}*\left( -i(2r-1-2l)\varphi_{i,0,1} \right) \right]$$

There will only be imaginary contributions which will not affect *R*. The second term in Eq. A9 will give the following,

$$-\omega_{i}g_{ii}\sum_{r=1}^{\infty} {(-1)}^{r}B_{r}\frac{(2r-1){R_{i,0,0}}^{2r-2}R_{i,0,1}}{2^{2r-1}}*\left[ \sum_{l=0}^{2r-1} {(-1)}^{l}\left( \begin{matrix} 2r-1 \\ l \end{matrix} \right)e^{-i\left( \left( 2r-2 \right)-2l \right)\left( \omega_{i}t \right)} \right]$$

The only contribution to *R* will be given by,

$${\omega_{i}}^{2}{g_{ii}}^{2}T\sum_{r=1}^{\infty} \left( -1 \right)^{r}B_{r}\frac{\left( 2r-1 \right){R_{i,0,0}}^{2r-2}}{2^{2r-1}}*\left[ \sum_{l=0}^{2r-1} \left( -1 \right)^{r-1}\left( \begin{matrix} 2r-1 \\ r-1 \end{matrix} \right) \right]*\sum_{s=1}^{\infty} \left( -1 \right)^{s}\left( -1 \right)^{s-1}B_{s}\frac{{R_{i,0,0}}^{2s-1}}{2^{2s-1}}\left( \begin{matrix} 2s-1 \\ s-1 \end{matrix} \right)$$

$$={\omega_{i}}^{2}{g_{ii}}^{2}T\sum_{r,s=1}^{\infty} B_{r}B_{s}\frac{\left( 2r-1 \right){R_{i,0,0}}^{2r+2s-3}}{2^{2r-1}2^{2s-1}}\left( \begin{matrix} 2r-1 \\ r-1 \end{matrix} \right)\left( \begin{matrix} 2s-1 \\ s-1 \end{matrix} \right)$$

1. $i=j\neq k$

When $i=j\neq k$ we will only get imaginary components which will not contribute to *R*.

1. $i\neq j=k$

When $i\neq j=k$ we will get only get imaginary components which will not contribute to *R*.

1. $i\neq j,j\neq k,i\neq k$

This condition ($i\neq j,j\neq k,i\neq k$) will be not contribute to *R* over a full cycle (*T*).

1. $i\neq j,i=k$

The RHS (Eq. A8) will give,

$$=-\omega_{i}\sum g_{ij}\sum_{r=1}^{\infty} {(-1)}^{r}B_{r}\frac{{R_{j,0,0}}^{2r-1}}{2^{2r-1}}*\left[ \sum_{l=0}^{2r-1} {(-1)}^{l}\left( \begin{matrix} 2r-1 \\ l \end{matrix} \right)e^{-i\left( \left( 2r-1 \right)-2l \right)\left( \omega_{j}t \right)+i\omega_{i}t}*\left( -i(2r-1-2l)\varphi_{j,1} \right) \right]-\omega_{i}\sum g_{ij}\sum_{r=1}^{\infty} {(-1)}^{r}B_{r}\frac{(2r-1){R_{j,0,0}}^{2r-2}R_{j,0,1}}{2^{2r-1}}*\left[ \sum_{l=0}^{2r-1} {(-1)}^{l}\left( \begin{matrix} 2r-1 \\ l \end{matrix} \right)e^{-i\left( \left( 2r-1 \right)-2l \right)\left( \omega_{j}t \right)+i\omega_{i}t} \right]$$

Again the terms contributing over a whole cycle will be imaginary and will not affect *R*.

In summary we have shown that potential-to-current coupling will not change the topology of the phase space structure, while the direct self-interaction terms of the current-to-current will.

**S1.3 Cross coupling between potential-to-current and current-to-current coupling**

It can be shown using a similar procedure to the above derivations that cross coupling between potential-to-current and current-to-current coupling will cause an instability in the phase space structure. In contrast, to what was shown in A1-2, off-diagonal terms ($g_{ij}, i\neq j$) will contribute to changes in R .

$$\frac{dR_{i,1,1}}{dt}=f(g_{ij},R_{i,0,0},R_{j,0,0},S,P)$$

**S1.4. Amplitude envelope dynamics**

The lead field of the EEG (Eq. 9) defines ellipsoidal surfaces in phase space where the amplitude power of the EEG will be constant, S1 Fig. As described in the main text we will perform a spatial average in phase space over these surfaces. The resultant spatially averaged amplitude will be a function of the distance to the origin.

**S1 Fig. 2D-ellipoids representing the surfaces with constant EEG amplitude.** The spatial average of the amplitude flow will be done over the positive sector (R_i_>0) of each of these surfaces.

Hyperspheroidal coordinates (in $\mathbb{R}^{4}$) can be used for the integration,

$$R_{1}= \frac{R}{a_{1}}cos\phi_{1}$$

$$R_{2}= \frac{R}{a_{2}}\sin\phi_{1}\cos\phi_{2}$$

$$R_{3}= \frac{R}{a_{3}}\sin\phi_{1}\sin\phi_{2}\cos\phi_{3}$$

$$R_{4}= \frac{R}{a_{4}}\sin\phi_{1}\sin\phi_{2}\sin\phi_{3}$$

The area unit for the ellipsoid is given by,

$$dE\equiv dE(\boldsymbol{a}, \boldsymbol{\phi)}$$

This is a complicated function of the coordinates ($\boldsymbol{\phi}$) and the components of the linear field ($\boldsymbol{a}$). Again, we will simplify to be able to integrate analytically. Instead of the ellipsoidal area unit we use the spheroidal area unit ($dA$), see S2 Fig. For a 3D-sphere this simplifies to,

$$dA= R^{3}\sin^{2}\phi_{1}\sin\phi_{2}d\phi_{1}d\phi_{2}d\phi_{3}$$

$$A= {\frac{1}{4}\pi^{2}R}^{3}$$

**S2 Fig. 2D-spherical and 2D-ellipsoidal area unit.** The spherical (yellow) and the ellipsoidal (blue) area unit are separated for better visualization. Using the spherical area unit instead of the elliptic will simplify the integrals considerably allowing for analytical solutions.

Note the following integral over $\frac{\pi}{2}$ :

$$\int\sin^{m}\phi_{1}{cos}^{n}\phi_{1}d\phi_{1}=PI=\frac{\sin^{m+1}\phi_{1}}{m+1}{cos}^{n-1}\phi_{1}+\frac{n-1}{m+1}\int\sin^{m+2}\phi_{1}{cos}^{n-2}\phi_{1}$$

$$\int\sin^{m}\phi_{1}{cos}^{n}\phi_{1}d\phi_{1}=\frac{\left( n-1 \right)‼\left( m-1 \right)‼}{\left( m+n-1 \right)‼}\int\sin^{m+n}\phi_{1}$$

The following terms need to be evaluated,

$$\int R_{i}\frac{dR_{i}}{dt}dA= \mu\omega_{i}g_{ii}\int\sum_{r=1}^{\infty} A_{r}\frac{{R_{i,0,0}}^{2r}}{2^{2r-1}}\left( \begin{matrix} 2r-1 \\ r-1 \end{matrix} \right)dA$$

We use the spherical area unit as an approximation to the ellipsoidal area unit to evaluate the requisite integrals. The self-interaction of the 1st population gives the following,

$$\mu\omega_{1}g_{11}\left( \begin{matrix} 2r-1 \\ r-1 \end{matrix} \right)\sum_{r=1}^{\infty} \frac{B_{r}}{2^{2r-1}}\int{R_{1,0,0}}^{2r}dA=\ldots\int{R_{1,0}}^{2r}dA$$

$$\int{R_{1,0,0}}^{2r}dA=\frac{R^{2r+3}}{{a_{1}}^{2r}}\int{cos}^{2r}\phi_{1}(1{-cos}^{2}\phi_{1})sin\phi_{2}d\phi_{1}d\phi_{2}d\phi_{3}$$

$$=\frac{R^{2r+3}}{{a_{1}}^{2r}}\int\cos^{2r}\phi_{1}-{cos}^{2r+2}\phi_{1}d\phi_{1}\int\sin\phi_{2}d\phi_{2}\int d\phi_{3}$$

Integral over $d\phi_{1}$,

$$\int\cos^{2r}\phi_{1}-{cos}^{2r+2}\phi_{1}d\phi_{1}=\frac{\pi}{2(2r+1)} \frac{\left( 2r+1 \right)‼}{\left( 2r+2 \right)‼}$$

Integral over $d\phi_{2}$,

$$\int\sin\phi_{2}d\phi_{2}=1$$

Integral over $d\phi_{2}$,

$$\int d\phi_{3}=\frac{\pi}{2}$$

Full integral

$$\int{R_{1,0}}^{2r}dA=\frac{4\pi^{2}R^{2r+3}}{(2r+1){a_{1}}^{2r}} \frac{\left( 2r+1 \right)‼}{\left( 2r+2 \right)‼}$$

From symmetry we get similar terms for the other populations.

$$\int R_{i}\frac{dR_{i}}{dt}dA= \mu\omega_{i}g_{ii}\sum_{r=1}^{\infty} B_{r}\frac{4\pi^{2}R^{2r+3}}{(2r+1)2^{2r}{a_{i}}^{2r}} \left( \begin{matrix} 2r-1 \\ r-1 \end{matrix} \right)\frac{\left( 2r+1 \right)‼}{\left( 2r+2 \right)‼}$$

Let $R_{s}$ be the average of *R* over the positive sector of the 3-sphere,

Eq. A10

$$\frac{dI_{s}}{dt}=\frac{1}{AR}∯ \sum{a_{i}}^{2}R_{i}\frac{dR_{i}}{dt}=\mu\sum{a_{i}}^{2} \omega_{i}g_{ii}\sum_{r=1}^{\infty} A_{r}\frac{4R^{2r-1}}{(2r+1)2^{2r}{a_{i}}^{2r}} \left( \begin{matrix} 2r-1 \\ r-1 \end{matrix} \right)\frac{\left( 2r+1 \right)‼}{\left( 2r+2 \right)‼}$$
