# Supplementary material for: Global dynamics of neural mass models
Source: PLoS Comput Biol. 2023 Feb 10;19(2):e1010915. doi: 10.1371/journal.pcbi.1010915 (PMC9949652; doi:10.1371/journal.pcbi.1010915)
Supplement: S2 Text — (DOCX) [file pcbi.1010915.s002.docx]

**S2 Text. Glossary**

This glossary list does not give exact mathematical definitions for the terms but is included to give an intuitive meaning for several of the technical terms in the paper.

Integral-differential equations – Equations that involve both integrals and derivatives of a function.

Partial differential equations – Equations that involve partial derivatives of a function.

2^nd^ order differential equations – Equations that involve the first and second derivatives of a function.

Mean-field approximation – Simplification of a model by averaging over degrees of freedom

Neural mass point processes – Mean-field approximation for a collection of interacting neural populations.

White noise – A random process with zero mean and fixed standard deviation.

Brownian noise – The effect of white noise interference on a stationary system.

Stochastic differential equation – Equations that involve derivatives and a noise process (e.g., white noise).

Fokker Planck equation – A partial differential equation whose solution describes how the probability density of states governed by a stochastic differential equation evolves with time.

Partition function – A function that describes the statistical properties of a system at equilibrium.

Sigmoid function – A function with a "S"-shaped graph.

Torus/tori – A set of geometric shapes, which in two dimensions has the shape of a doughnut.

Hyperspheroidal coordinates – A coordinate system for spheres, which in two dimensions is given by the longitude, latitude and radius of a point on a sphere.

Modulus-argument – The length of the line segment of a complex number is called the modulus. The angle measured from the positive real axis to the line segment is called the argument of the complex number.

Phase space representation – It is a geometric description of a system, where all the possible states of the system are represented by unique points. The dynamics of the system are described by trajectories in phase space.

Attractor – Set of points in phase space that attracts trajectories. A chaotic attractor has a complex geometric structure.

Stationary points – Points of equilibrium in phase space.

Limit cycle – A trajectory in phase space which is a closed loop with at least one other trajectory approaching it as time approaches infinity or negative infinity.

Gradient flow – A “force” in phase space that is given by the derivative of a scalar function (potential function).
