# Supplementary figures and images for: Global dynamics of neural mass models
Source: PLoS Comput Biol. 2023 Feb 10;19(2):e1010915. doi: 10.1371/journal.pcbi.1010915 (PMC9949652; doi:10.1371/journal.pcbi.1010915)

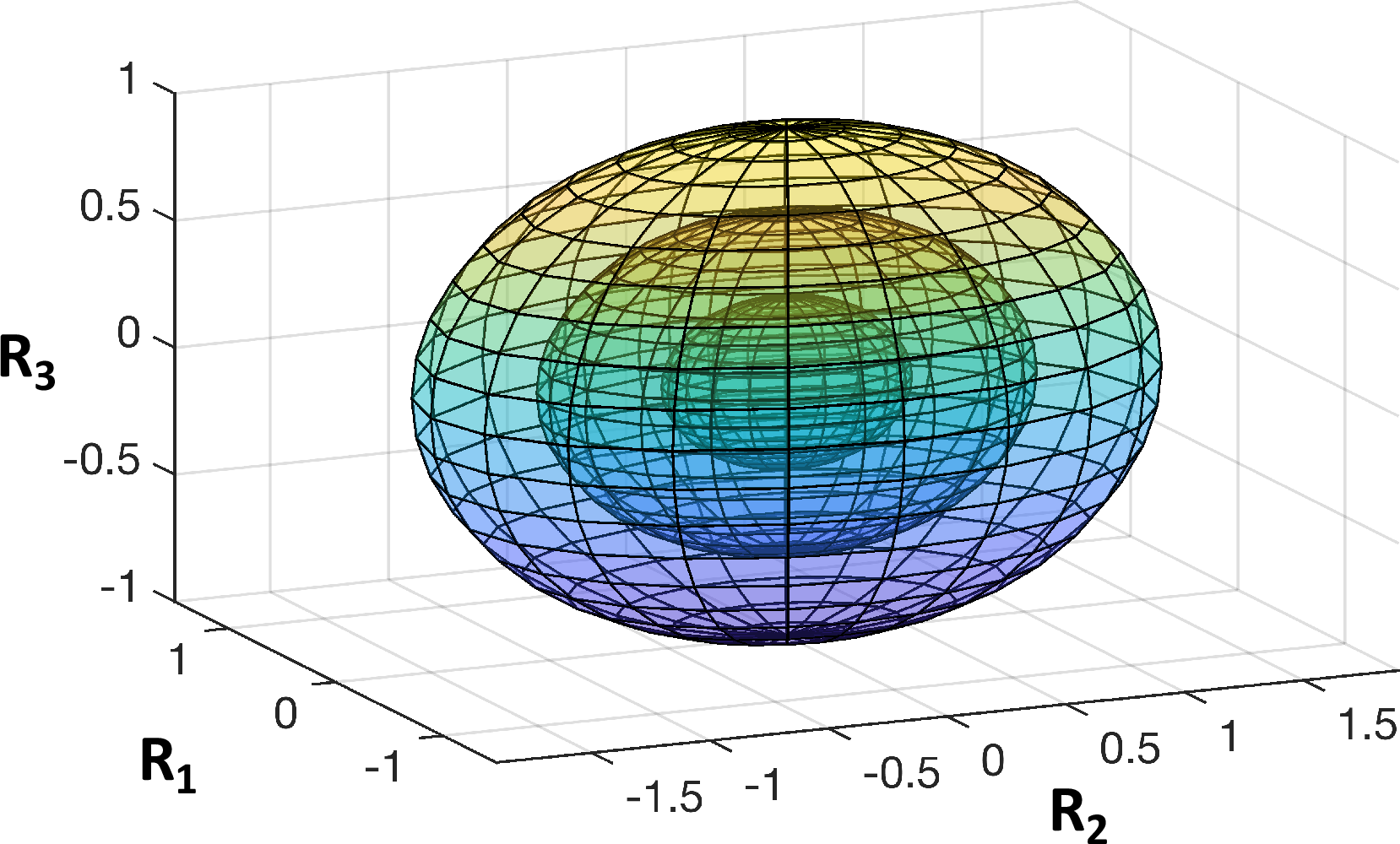

Supplement: S1 Fig — The spatial average of the amplitude flow will be done over the positive sector (Ri>0) of each of these surfaces. (TIF) [file pcbi.1010915.s003.tif]

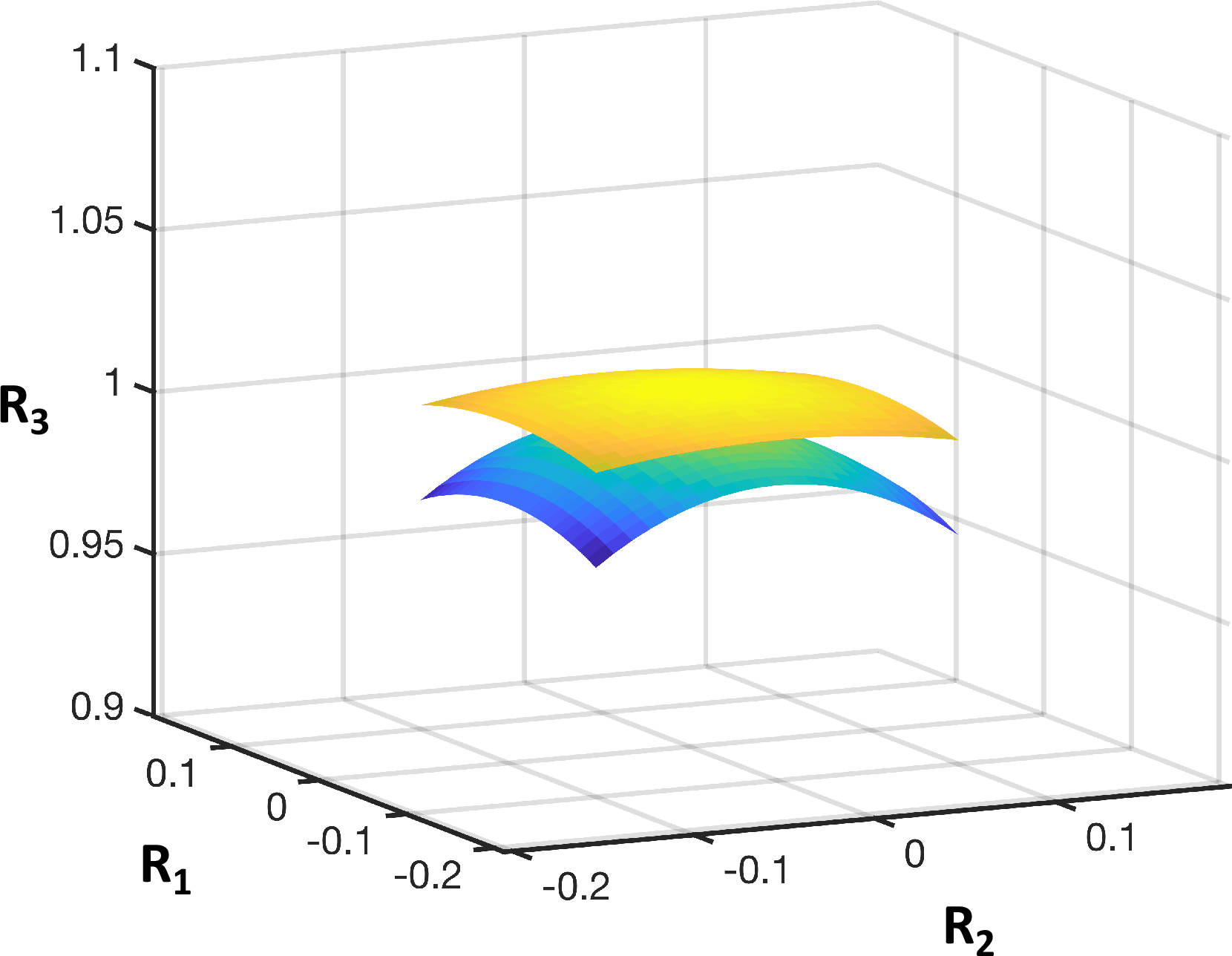

Supplement: S2 Fig — The spherical (yellow) and the ellipsoidal (blue) area unit are separated for better visualization. Using the spherical area unit instead of the elliptic will simplify the integrals considerably allowing for analytical solutions. (TIF) [file pcbi.1010915.s004.tif]
