# Supplementary material for: Global dynamics of neural mass models
Source: PLoS Comput Biol. 2023 Feb 10;19(2):e1010915. doi: 10.1371/journal.pcbi.1010915 (PMC9949652; doi:10.1371/journal.pcbi.1010915)
Supplement: Attachment — Submitted filename: review letter 20230106_KF.docx [file pcbi.1010915.s005.docx]

Reviewer #1: In their paper, Cooray et al. made a thorough analysis of a popular and effective type of brain dynamics models, i.e. neural mass models, which are a coarse-grained version of neural field models that has shown its relevance in many applications (e.g., epilepsy). The paper has the merit of proposing a detailed mathematical analysis that describes the behavior of those models not only around the fixed points, which is useful to understand the dynamical repertoire of those models.

1) The paper is difficult to follow for a non-specialist. The paper makes an extensive use of technical terms borrowed from different sub-fields of physics (dynamical itinerancy, adiabatic approximation, potential function, equations of motion...) which complexifies reading in an unnecessary manner. Extensive editing should improve this aspect to make the paper more accessible and increase its impact. Technical terms such as those, when unavoidable, should be defined operationally to facilitate reading of the paper.

Reply: Thank you for these recommendations. We have now rewritten the paper such that it is accessible to a wider audience but keeping detailed descriptions where necessary to allow for the analysis to be transcribed into software. We have also included a glossary for a description of technical terms.

We have now added a glossary list to the supplemental section:

“S2 Glossary

This glossary list does not give exact mathematical definitions for the terms but is included to give an intuitive meaning for several of the technical terms in the paper.

Integral-differential equations – Equations that involve both integrals and derivatives of a function.

Partial differential equations – Equations that involve partial derivatives of a function.

2^nd^ order differential equations – Equations that involve the first and second derivatives of a function.

Mean-field approximation – Simplification of a model by averaging over degrees of freedom

Neural mass point processes – Mean-field approximation for a collection of interacting neural populations.

White noise – A random process with zero mean and fixed standard deviation.

Brownian noise – The effect of white noise interference on a stationary system.

Stochastic differential equation – Equations that involve derivatives and a noise process (e.g., white noise).

Fokker Planck equation – A partial differential equation whose solution describes how the probability density of states governed by a stochastic differential equation evolves with time.

Partition function – A function that describes the statistical properties of a system at equilibrium.

Sigmoid function – A function with a "S"-shaped graph.

Torus/tori – A set of geometric shapes, which in two dimensions has the shape of a doughnut.

Hyperspheroidal coordinates – A coordinate system for spheres, which in two dimensions is given by the longitude, latitude and radius of a point on a sphere.

Modulus-argument – The length of the line segment of a complex number is called the modulus. The angle measured from the positive real axis to the line segment is called the argument of the complex number.

Phase space representation – It is a geometric description of a system, where all the possible states of the system are represented by unique points. The dynamics of the system are described by trajectories in phase space.

Attractor – Set of points in phase space that attracts trajectories. A chaotic attractor has a complex geometric structure.

Stationary points – Points of equilibrium in phase space.

Limit cycle – A trajectory in phase space which is a closed loop with at least one other trajectory approaching it as time approaches infinity or negative infinity.

Gradient flow – A “force” in phase space that is given by the derivative of a scalar function (potential function).”

2) The potential applications listed are not sufficiently described, which is not convincing: the authors should insist on why their approach will provide new insights in epilepsy (where much has been done in terms of neural mass modeling / analysis, more on this point below) at the onset of seizures, or for the description of beta bursts in Parkinson's disease. In its current form, this gives the impression that the authors have just listed some applications but without any specific reason or rationale (and there is likely one I am sure, but it is not obvious in the current form of the paper).

Reply: Thank you for these comments. We have now re-organized and re-written the paper such that the reason and rationale for the study is foregrounded. Furthermore, we have made it clear what the paper brings to the understanding of epilepsy.

We now write:

Introduction

“The theoretical and empirical applications of neural mass models — with separation of time scales — to analyze electrophysiological data, especially seizure activity, are diverse [28-33]. A seminal paper, by Jirsa et al., elegantly modelled epileptic seizure activity, in terms of bifurcations, using a neural mass model with two neural masses and a “permittivity” variable which introduced a separation of time scales. The slow permittivity variable reflects chemical changes, such as variations in electrolyte or metabolic homeostasis. This work has further been elaborated in computational pipelines that can be applied to specific patient data recorded in the evaluation for epilepsy surgery [34]. The Epileptor model differs from the canonical micro-circuit model, as the latter does not have explicit multiple time scales. However, we show in this paper that neural mass models do have an implicit time scale variation, which could explain the phenomena described by the Epileptor model. The method we use to study the implicit time scales of neural mass models are standard techniques in the study of dynamical systems [35, 36] and was alluded to by the authors of the Epileptor model [37]. “

Results

“This work could be applied when investigating spiking in epileptic and non-epileptic tissue as the inter-spike interval could be modelled as the exit time from a large amplitude transient. The inversion method described could then map spike frequency to synaptic connectivity, which could be used to uncover the underlying physiological differences between regions of the brain with different epileptogenicity.”

3) The authors mention that one innovation of their approach is the decomposition of the classical neural mass model (that they call "Canonical Microcircuit Model" or CMC) in terms of slow/fast sub-systems, and bifurcation analysis. However, slow-fast decomposition of the CMC, notably in the field of epilepsy, has been already performed previously (see for example this recent study uncited by the authors, <https://doi.org/10.1371/journal.pcbi.1008430>, which performs slow/fast decomposition at the initiation of seizures). Therefore, the innovation brought by the paper is unclear, and should be emphasized to clarify the potential impact of the paper and its interest for a broad readership.
4) It seems that previous literature (such as on the Epileptor model, <https://doi.org/10.1093/brain/awu133>) should be more discussed or taken into account, given the overlap with the present work.

Reply: Thank you for pointing this out and the recommendation for citations. We have now placed the work of this paper in context of current research, again underscoring what we bring to the understanding of the electrophysiology of seizures. We have also included the above references and several others.

We now say, in the Discussion

“It is important to compare this work with other published methods using neural mass models to investigate itinerant brain dynamics. The Epileptor model is frequently used to characterize epileptic seizure onset, propagation and offset [28, 34, 66]. As described previously, the timescales are explicit in the functional form of the equations of the Epileptor model; while they are only implicit in the physiologically grounded (canonical microcircuit) model described in this study. The explicit parameterization in terms of distinct rate (i.e., time) constants simplifies the mathematical modelling of seizures and provides a compelling taxonomy of seizures in terms of bifurcations for the field of computational epilepsy. The CMC-model, in contrast, requires extensive mathematical derivations to uncover the underlying slow processes; however, the biophysics of slow modulation can be shown to depend on the synaptic connectivity within a cortical column. This may be important in terms of understanding the mechanisms (e.g., synaptopathies) that underwrite epilepsy.

We have thus shown the possibility of a cortical column evincing slow dynamics in the absence of slowly fluctuating variables. This dynamical mechanism fits well with the principle of self-organization, where a system shows patterns in time or spatial scale larger than the local dynamics governing the system. The analysis provided in this paper further allows for the statistics of transient electrophysiological activity (e.g., spike frequency, seizure duration, burst duration in Parkinson’s disease) to be mapped onto the synaptic connectivity, in contrast—or as a complement—to the permittivity variable in the Epileptor model.

Finally, there is a key difference between accounts exemplified by the Epileptor model, and the model described in this paper. We describe a causal flow from fast to slow timescales, in contrast to a feed-back system of the kind found in the Epileptor model. There is an “arrow of causality” in the present work that is similar to that found in thermodynamics and physics in general, from microscopic to macroscopic [68-72]. However, we note that the feed-back from slow to fast timescales may be required in certain cases (e.g., periodic seizures or seizures of longer duration), perhaps using a similar set-up as the Epileptor model [28]. This speaks to some interesting issues about the nature of circular causality, and bottom-up versus top-down causation [66,73]. These issues could, in principle, be addressed using Bayesian model comparison of appropriate dynamic (causal) models. ”

“This form of transition between states has been described in terms of transcritical activity [39]. What is new in this study is that we can now map the observed changes to synaptic connectivity within the cortex.”

**Have the authors made all data and (if applicable) computational code underlying the findings in their manuscript fully available?**

Reviewer #1: **No:** Only general websites are listed, the information provided to access the code / data is not sufficiently specific.

Reply: No problem. We have now placed the codes/simulated data for the paper on GitHub, https://github.com/gercoo/exittimes2synapticconnectivity

Reviewer #2: In this paper, the authors present a novel, elegant, and useful way to reduce the complexity of neural mass models of local cortical microcircuits by means of time scale separation, thus making them more accessible to model inversion, especially in larger portions of the state space away from fixed points. Although, personally, I would have liked a somewhat more elaborate evaluation of the methods using simulations and real data, I think the paper is worth publishing as it stands. The paper is very well written, so I have no further comments (which is a very rare case for me).

Reply: Thank you very much for your kind comments. We are preparing a new study with applications of the methods described in this study to real data. Specifically, we hope to analyze retrospective data from pediatric epilepsy, to see if the theory reported in this paper provides models with some predictive validity. This work is currently ongoing and will refer to the current paper as its theoretical foundation.
