# Supplementary material for: Global dynamics of neural mass models
Source: PLoS Comput Biol. 2023 Feb 10;19(2):e1010915. doi: 10.1371/journal.pcbi.1010915 (PMC9949652; doi:10.1371/journal.pcbi.1010915)
Supplement: Attachment — Submitted filename: Responses to Reviewer comments 20230131.docx [file pcbi.1010915.s006.docx]

**Responses to Reviewer comments**

Editorial Note: Please make a final read and edit of the paper, ensuring where possible a clear integration of the technical terms in the main text and subheadings with the glossary as per the reviewer's request

Reviewer's Responses to Questions

**Comments to the Authors:
Please note here if the review is uploaded as an attachment.**

Reviewer #1: The authors have provided a revision that partly addresses the comments raised in the first review. The addition of an appendix with a lay definition of technical terms is very much welcome, however within the body of the paper, nothing has changed much in this regards, as is apparent in the "Track changes" version of the document. Therefore, the paper remains filled, including in the Section titles, with terms that are as I mentioned before unnecessarily complicated.

Response: Thank you for the comments. We have now referenced the technical terms (on first occurrence) in the main text to the glossary and removed technical terms from the section titles. Furthermore, we have simplified the introduction to increase accessibility and use technical terms in the method and result section as required for completeness.

Furthermore, on the topic of Parkinson's disease, what has been added is very vague, and only "bursts" are mentioned: in which region, at rest, during tremor, during a task? Furthermore, no reference is provided at all. I would suggest either to suppress Parkinson's disease from the paper, or to add precise and referenced content. The relevance of citing PD would also be an asset. Otherwise, the authors may as well cite almost any disease.

Response: Thank you for bringing this up; we have now removed discussion of PD from the paper.

Also, the "arrow of causality" term is one of those terms that is technical for a large portion of the readership, should be defined, and also should be supported clearly by the results. If the authors want to keep such a term in the paper, this would deserve explaining this in more details.

Response: Thank you for this comment. We have now defined the term together with supporting evidence from the results.

We now write,

“Finally, there is a key difference between accounts exemplified by the Epileptor model, and the model described in this paper. The dynamics of slow variables (e.g. amplitude modulation) derived in this paper are dependent on fast variables (e.g. high frequency activity within neural populations) only via the adiabatic approximation. In other words, the fast activity pushes the system configuration slowly giving rise to the implicit slow dynamics. We describe a causal flow from fast to slow timescales, in contrast to a feed-back system of the kind found in the Epileptor model, where the dynamics of the slow variables directly affect faster variables. The dependence of slow variables on fast variables in this paper allows us to define an “arrow of causality” that is similar to that found in physics, where macroscopic variables can be estimated from the underlying microscopic variables but not vice versa. [68-72].”

**Have the authors made all data and (if applicable) computational code underlying the findings in their manuscript fully available?**
The [PLOS Data policy](https://eur01.safelinks.protection.outlook.com/?url=https%3A%2F%2Fjournals.plos.org%2Fploscompbiol%2Fs%2Fmaterials-and-software-sharing&data=05%7C01%7Cgerald.cooray%40ki.se%7C905e0ef842cb4ddd51ac08db003a5912%7Cbff7eef1cf4b4f32be3da1dda043c05d%7C0%7C0%7C638104022823253927%7CUnknown%7CTWFpbGZsb3d8eyJWIjoiMC4wLjAwMDAiLCJQIjoiV2luMzIiLCJBTiI6Ik1haWwiLCJXVCI6Mn0%3D%7C3000%7C%7C%7C&sdata=4AFCzL%2Bj39cQsYO5ganLO%2FYifFfb0nu4uI0%2FQ7da0%2BQ%3D&reserved=0) requires authors to make all data and code underlying the findings described in their manuscript fully available without restriction, with rare exception (please refer to the Data Availability Statement in the manuscript PDF file). The data and code should be provided as part of the manuscript or its supporting information, or deposited to a public repository. For example, in addition to summary statistics, the data points behind means, medians and variance measures should be available. If there are restrictions on publicly sharing data or code —e.g. participant privacy or use of data from a third party—those must be specified.

Reviewer #1: Yes

PLOS authors have the option to publish the peer review history of their article ([what does this mean?](https://eur01.safelinks.protection.outlook.com/?url=https%3A%2F%2Fjournals.plos.org%2Fploscompbiol%2Fs%2Feditorial-and-peer-review-process%23loc-peer-review-history&data=05%7C01%7Cgerald.cooray%40ki.se%7C905e0ef842cb4ddd51ac08db003a5912%7Cbff7eef1cf4b4f32be3da1dda043c05d%7C0%7C0%7C638104022823253927%7CUnknown%7CTWFpbGZsb3d8eyJWIjoiMC4wLjAwMDAiLCJQIjoiV2luMzIiLCJBTiI6Ik1haWwiLCJXVCI6Mn0%3D%7C3000%7C%7C%7C&sdata=MTmQuvE6JaKpoTSkCM9Z3cn6omIN1yVDcmDzlhq3IjY%3D&reserved=0)). If published, this will include your full peer review and any attached files.


If you choose “no”, your identity will remain anonymous but your review may still be made public.


**Do you want your identity to be public for this peer review?** For information about this choice, including consent withdrawal, please see our [Privacy Policy](https://eur01.safelinks.protection.outlook.com/?url=https%3A%2F%2Fwww.plos.org%2Fprivacy-policy&data=05%7C01%7Cgerald.cooray%40ki.se%7C905e0ef842cb4ddd51ac08db003a5912%7Cbff7eef1cf4b4f32be3da1dda043c05d%7C0%7C0%7C638104022823253927%7CUnknown%7CTWFpbGZsb3d8eyJWIjoiMC4wLjAwMDAiLCJQIjoiV2luMzIiLCJBTiI6Ik1haWwiLCJXVCI6Mn0%3D%7C3000%7C%7C%7C&sdata=FS7Hll59CwoOdAeAqFeNgmTJvx98p7g0ncXS4iHp0SI%3D&reserved=0).

Reviewer #1: No

Figure Files:

While revising your submission, please upload your figure files to the Preflight Analysis and Conversion Engine (PACE) digital diagnostic tool, <https://pacev2.apexcovantage.com>. PACE helps ensure that figures meet PLOS requirements. To use PACE, you must first register as a user. Then, login and navigate to the UPLOAD tab, where you will find detailed instructions on how to use the tool. If you encounter any issues or have any questions when using PACE, please email us at [figures@plos.org](mailto:figures@plos.org).

Data Requirements:

Please note that, as a condition of publication, PLOS' data policy requires that you make available all data used to draw the conclusions outlined in your manuscript. Data must be deposited in an appropriate repository, included within the body of the manuscript, or uploaded as supporting information. This includes all numerical values that were used to generate graphs, histograms etc.. For an example in PLOS Biology see here: <http://www.plosbiology.org/article/info%3Adoi%2F10.1371%2Fjournal.pbio.1001908#s5>.

Reproducibility:

To enhance the reproducibility of your results, we recommend that you deposit your laboratory protocols in protocols.io, where a protocol can be assigned its own identifier (DOI) such that it can be cited independently in the future. Additionally, PLOS ONE offers an option to publish peer-reviewed clinical study protocols. Read more information on sharing protocols at [https://plos.org/protocols?utm_medium=editorial-email&utm_source=authorletters&utm_campaign=protocols](https://eur01.safelinks.protection.outlook.com/?url=https%3A%2F%2Fplos.org%2Fprotocols%3Futm_medium%3Deditorial-email%26utm_source%3Dauthorletters%26utm_campaign%3Dprotocols&data=05%7C01%7Cgerald.cooray%40ki.se%7C905e0ef842cb4ddd51ac08db003a5912%7Cbff7eef1cf4b4f32be3da1dda043c05d%7C0%7C0%7C638104022823253927%7CUnknown%7CTWFpbGZsb3d8eyJWIjoiMC4wLjAwMDAiLCJQIjoiV2luMzIiLCJBTiI6Ik1haWwiLCJXVCI6Mn0%3D%7C3000%7C%7C%7C&sdata=%2BcMtCFw6it0NGGqta2tqREKDkncLbBoweyxcQCY0V4U%3D&reserved=0)

References:

Review your reference list to ensure that it is complete and correct. If you have cited papers that have been retracted, please include the rationale for doing so in the manuscript text, or remove these references and replace them with relevant current references. Any changes to the reference list should be mentioned in the rebuttal letter that accompanies your revised manuscript.

*If you need to cite a retracted article, indicate the article’s retracted status in the References list and also include a citation and full reference for the retraction notice.*
